# Cognitive Writing Process Characteristics in Alzheimer’s Disease

**DOI:** 10.3389/fpsyg.2022.872280

**Published:** 2022-07-11

**Authors:** Catherine Meulemans, Mariëlle Leijten, Luuk Van Waes, Sebastiaan Engelborghs, Sven De Maeyer

**Affiliations:** ^1^Research Foundation – Flanders, Brussels, Belgium; ^2^Department of Management, University of Antwerp, Antwerp, Belgium; ^3^Department of Biomedical Sciences, University of Antwerp, Antwerp, Belgium; ^4^Center for Neurosciences (C4N), Vrije Universiteit Brussel, Brussels, Belgium; ^5^Department of Neurology, Universitair Ziekenhuis Brussel, Brussels, Belgium; ^6^Department of Neurology and Memory Clinic, Hospital Network Antwerp (ZNA) Middelheim and Hoge Beuken, Antwerp, Belgium; ^7^Department of Training and Education Sciences, University of Antwerp, Antwerp, Belgium

**Keywords:** writing processes, word categories, keystroke logging, Alzheimer’s disease, dementia, mild cognitive impairment

## Abstract

In this article, we explore if the observation of writing behavior can assist in the screening and follow-up of mild cognitive impairment (MCI) and mild dementia due to Alzheimer’s disease (AD). To this end, we examined the extent to which overall writing process measures and pausing behavior during writing differed between 15 cognitively impaired patients and 15 age- and gender-matched healthy controls. Participants completed two typed picture description tasks that were registered with Inputlog, a keystroke logging program that captures keyboard activity during text production. The following variables were analyzed with mixed-effects models: time on task; number of characters, pauses and Pause-bursts per minute; proportion of pause time; duration of Pause-bursts; and pause time between words. For pause time between words, also the effect of pauses preceding specific word categories was analyzed. Results showed a main effect of group on all variables. In addition, for pause time between words a main effect of part-of-speech was found as well. Results indicate that writing process analysis can possibly serve as a supplementary tool for the screening and follow-up of AD.

## Introduction

Patients with Alzheimer’s disease (AD) show a progressive cognitive decline that includes language comprehension and production (Blazer and Steffens, 2009; Ferguson et al., 2014). Findings from the Nun Study, for example, showed that longitudinal changes in linguistic ability, reflected by idea density, were strongly related to dementia and AD in later life (Riley et al., 2005). Researchers like Garrard et al. (2005); Le et al. (2011), and Marckx et al. (2018) also showed that the work of novelists with AD (e.g., Iris Murdoch or Hugo Claus), was characterized by a linguistic decline that clearly differed from that related to healthy aging. Narratives of healthy aging adults have shown signs of language decline as well. For example, in a study by Kemper (1990) older adults’ more frequent use of ambiguous anaphors resulted in less coherent texts. Aging is also associated with a loss of idea density and syntactic complexity, the latter reflected by the use of shorter sentences and fewer embedded and non-embedded constructions (Kemper, 1987). However, some studies do suggest that language changes become more pronounced in AD than in healthy aging (Kemper et al., 2001).

The above implies that written discourse analysis may be quite sensitive in detecting subtle deficits in cognitive status, and may even be more suitable than speech for distinguishing impaired from healthy elderly (Mitzner and Kemper, 2003). In addition, even though most studies only analyze the final texts, the writing processes leading up to those texts could also provide valuable information on cognition. For example, if someone is having difficulties forming a sentence or naming an item, this might already be reflected in the writing process (e.g., in terms of latencies) rather than in the final text.

### Spoken Versus Written Language Production in Aging and Alzheimer’s Disease Studies

A number of studies examined differences between speech and writing associated with aging (Croisile et al., 1996; Mitzner and Kemper, 2003; Forbes-McKay et al., 2013, 2014). Croisile et al. (1996) compared oral and written descriptions of a picture commonly used in the assessment of aphasia [the Cookie Theft picture (Goodglass et al., 2001)]. Of the 22 patients with probable AD and their 24 matched controls, patients produced fewer words across all word categories, fewer predefined information units (also called pictorial themes), and sentences that were grammatically less complex (e.g., fewer subordinate clauses). For either group, no significant difference between oral and written modalities was found in the use of pictorial themes. However, the increase of irrelevant information and the decrease in syntactic complexity in the written task led the researchers to conclude that the written mode was more sensitive for identifying linguistic difficulties present in AD.

Forbes-McKay et al. (2013, 2014) also conducted a cross-sectional and longitudinal study on speech and writing to document the progression of linguistic impairments in AD over a 12-month period. Thirty-one probable AD patients and 30 matched controls completed a simple and a complex narrative description task orally and through handwriting. Cross-sectional comparisons indicated that the language of mild-moderate AD patients was characterized by a higher number of empty and indefinite phrases, fewer pictorial themes and error corrections, and shorter and grammatically more simplified sentences in both the oral and the written mode. Follow-up data suggested that both phonological and visual processing declined over time. In these studies the data were analyzed manually.

Toledo et al. (2014) added an automated component to these analyses. They used a simple (“The Tripping Woman Picture”) and a complex line drawing (“The Traffic Chaos Picture”) that were also used by Forbes-McKay et al. (2013, 2014). Their first goal was to develop machine learning classifiers to classify healthy subjects based on years of education. For this, linguistic features, such as words per sentence and incidence of nouns, verbs and adjectives, were extracted from handwritten descriptions with the use of natural language processing tools. Their second goal was to automatically identify the features that were most suited to distinguish the groups. Results showed that, for data classification, a specific type of machine learning (i.e., Support Vector Machine with a radial basis function kernel) performed best. Moreover, correlation-based feature selection was the preferred method to replace manual selection with (Toledo et al., 2014). These findings also support the idea that groups can be distinguished on the basis of specific linguistic properties.

### Typing Skills and Writing

Not only written texts (product), but also keyboard interactions (process) can indicate cognitive (and motoric) status. For example, machine learning research has shown that keyboard typing can detect cognitive stress (Vizer et al., 2009; Unni et al., 2021). In a study by Vizer et al. (2009) keystroke features (e.g., time per keystroke) and linguistic features (e.g., lexical diversity) of free text production were identified. When used to classify a cognitive stress condition relative to a non-stress condition the classification accuracy amounted to 75% (i.e., a percentage also obtained in affective computing methods). This indicates that keyboard interactions might be able to track changes in cognitive stress related to both aging and AD.

This is supported by copy typing studies, such as a study by Van Waes et al. (2021) that showed a non-linear relationship between typing speed and age. Participants from 13 to 83 years old performed a copy task in which they had to copy type letters, sentences and words from a screen. The non-linearity was present for all subtasks, but differed for lexical (e.g., typing of sentences) compared to non-lexical subtasks (e.g., tapping task). Overall, participants typed fastest between the ages of 21 and 30, but after that their speed started to decrease. In addition, subtasks with lexical components showed a gradual increase in typing speed between the ages of 13 and about 25, while this was not the case for non-lexical subtasks. In the older age groups (from about 50 years old), typing speed also decreased more in lexical than in non-lexical subtasks (Van Waes et al., 2021). Another study with the same copy task showed that keyboard typing can also detect gradual changes in motor performance related to AD (Van Waes et al., 2017). Young adults, healthy elderly and age-matched impaired elderly performed the copy task. Results showed not only a decrease in typing speed with increasing age (i.e., between young adults and healthy elderly), but also a slowdown for cognitively impaired elderly compared to healthy elderly. These observations suggest that aging affects the skills required for lexical processing and further support the idea that a typing task could be promising for screening and follow-up of Alzheimer patients − especially because the type of task allows to measure a combination of motor and cognitive capacities in language production (Van Waes et al., 2017, 2021).

### Writing Dynamics and Pausing Behavior

In writing and in speech, pausing behavior has proven to be an important resource for identifying cognitive effort during text composition (Wengelin, 2006; Vizer et al., 2009). Pausing behavior points to production flow interruptions [the term often used here is “fluency” (Hayes and Chenoweth, 2007; Van Waes and Leijten, 2015)]. This can be measured, for instance, in terms of the number and length of pauses. According to capacity theory, interruptions result from exceeding the limited capacity of working memory (Just and Carpenter, 1992; McCutchen, 1996). The large number of concurrent writing processes that take place during writing cause a cognitive overload. Subsequently, the writer pauses so that working memory resources are freed up and can be used for other, more demanding processes (e.g., planning, formulating or reviewing). Once enough processes are completed, writing can resume (Kellogg, 2001; Olive and Kellogg, 2002; Olive, 2014).

In tandem with pauses, also the number and length of production bursts change with cognitive effort. When the number of parallel processes reduces, the writing process shows more fragmentation (i.e., more switching between pausing and writing), characterized by longer pauses and shorter bursts. When the number of parallel processes increases, this pattern changes to less fragmentation with shorter pauses and longer bursts. This was demonstrated by studies examining the influence of transcription automation on the distribution of pauses and bursts during writing. The cognitive load of non-automatised transcription can, in fact, be quite high. Studies in children and adults showed that writing processes are less fluent when typing is less automated, which is reflected by more and longer pauses and shorter bursts (Alves et al., 2007; Alves and Limpo, 2015; Leijten et al., 2019).

In healthy aging, these disfluencies are especially evident in the form of longer pause times caused by word retrieval failures such as tip-of-the-tongue states (Burke and Shafto, 2004; Verhaegen and Poncelet, 2013). This decline in working memory capacity with age was also suggested by studies using a copy task; a task that minimizes the load of cognitive processes such as planning, formulating and reviewing on working memory. A study by Van Waes et al. (2021) showed higher interkey latencies for older participants. In addition, the influence of age was more pronounced in lexical than in non-lexical tasks, an effect that was likely caused by the added load of formulating on working memory (Kliegel and Jäger, 2006; Wild-Wall et al., 2011; Van Waes et al., 2021). With regards to AD, patients tend to be less fluent writers as well, reflected by shorter production bursts and more pauses. Moreover, they pause longer before and within words than their healthy counterparts (Leijten et al., 2015).

#### Measuring Pausing Behavior

**FIGURE 1:**
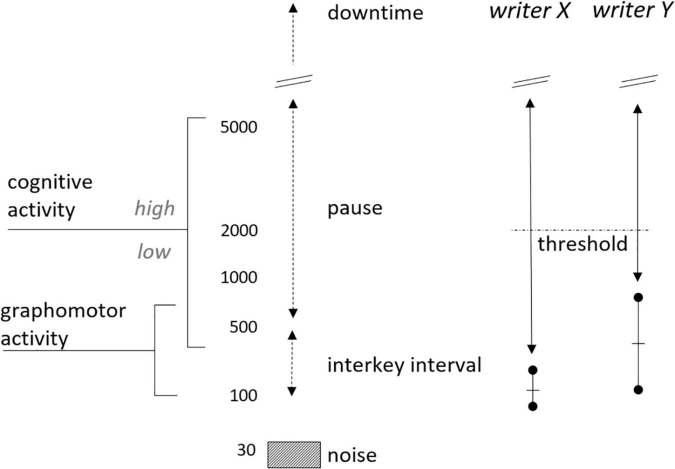
Schematic representation of latency in relation to graphomotor activity, and low- and high-level cognitive activities. From Van Waes et al. (2021). Reprinted with permission.

Since a certain amount of time is always required to move from one key on the keyboard to the next, not every moment without transcription can be considered a pause. A first approach to correctly define pauses is to apply a pause threshold to those transition times (or interkey intervals). Only interkey intervals above that specific threshold are then considered actual pauses. In target groups where typing is more fluent, transitioning between two keys is in general faster than the pause threshold of 2 s that is used in quite some writing studies. A lower threshold (i.e., 200 ms) could therefore be more appropriate for, for example, a group of students who can touch type (Wengelin, 2006). Moreover, choosing a threshold also means opting for a certain data filtering and, as a result, studying the cognitive processes related to those filtered data. With a higher pause threshold longer pause times will remain, which are mainly associated with high-level cognitive processes, like planning new content or preparing a (substantial) revision. In contrast, a lower pause threshold also includes shorter pauses, which are mainly associated with low-level cognitive processes, like word transitions or typo corrections (see Figure 1; Van Waes and Leijten, 2015).

Moreover, differences in motoric speed do not only occur between target groups, but also between individuals of those groups. In a target group that consists of participants with different typing skills, chances are small that one pause threshold applies to each individual in that group. A second approach is therefore to set a separate threshold for each person, which can be regarded as each person’s personal typing speed. These personal thresholds are often determined on the basis of copy typing studies in which participants are instructed to type a given text, sentence or word combination as quickly as possible. Because the text is predetermined, the involvement of writing (sub)processes is minimized and participants’ working memory resources are mainly used for transcription. Transition times are then considered to reflect mainly motoric typing speed and allow for the calculation of a personal threshold per participant that can be used as a covariate in pause analyses (Van Waes et al., 2019, 2021). The distinction between a transition time and an actual pause also shows that the writing process has both a cognitive and a motor component (see Figure 1). This means that language change can be approached from these two complementary angles.

#### Linguistic Aspects in Writing Processes

A study comparing fluency in a first language (L1; Dutch) and a second language (L2; English) found a link between cognitive effort, measured in pause times, and specific linguistic properties. Writing process analyses showed higher pauses between words for L2 than for L1, indicating the dependence of pause times on participants’ familiarity with a language. A main effect of parts-of-speech and an interaction effect between language and parts-of-speech showed that pause times also varied depending on the word category they belonged to (e.g., nouns or verbs), thereby illustrating the importance of analyzing writing processes from a linguistic standpoint (Leijten et al., 2019).

In other studies, linguistic aspects have also proven to mediate pause occurrence and length. Firstly, pause time is affected by pause location: pauses before lexical units at higher text levels (e.g., paragraphs) are longer than those at lower levels (e.g., words, syllables, or characters) (Wengelin, 2006; Medimorec and Risko, 2017). Secondly, linguistic aspects adhering to those lexical units influence pauses. For example, the grammatical category a word belongs to affects that word’s initial pause length (onset). Studies on action and object naming with healthy participants often indicated that verbs are more difficult to produce than nouns and, therefore, evoked longer naming latencies (Bogka et al., 2003; Szekely et al., 2005; Druks et al., 2006; Mätzig et al., 2009). However, results of studies on AD patients tend to vary more. Some indicated more difficulties with verbs than with nouns, while others found the opposite (Fung et al., 2001; Druks et al., 2006). In addition, production of other word categories (e.g., adjectives) is, to our knowledge, largely left unstudied, both in healthy and cognitively impaired subjects.

### Alzheimer’s Disease Screening

Research into the development of language-based screening instruments for AD is scarce and rather limited in scope. First, it almost exclusively examines the characteristics and progression of cognitive decline on the basis of product measures (i.e., based on the final texts; McGeown et al., 2009; Forbes-McKay et al., 2013, 2014; Toledo et al., 2014). This is also reflected in most studies focusing on writing in elderly, such as those focused on text analysis of healthy elderly (e.g., Kemper, 1990; Ferguson et al., 2014) or of people with AD (e.g., Snowdon et al., 1996; Garrard et al., 2005; Pakhomov et al., 2011; Forbes-McKay et al., 2014). In this paper, we want to add to these studies by exploring the possibilities of writing *process* dynamics to identify cognitive decline.

Second, language-related assessment tools in the diagnostic work-up of AD have hardly changed over the last 30 years. However, the information technology landscape has changed considerably. Especially the increase of digitalization in written text production is remarkable (Brandt, 2014), and the group of elderly with computer literacy is continuously growing (Jones and Fox, 2009; Zhou, 2011). This paper will therefore look at typed rather than handwritten texts. An instrument that can detect cognitive decline based on digital writing processes (i.e., captured with keystroke logging) would be easy to apply, cheap, unobtrusive, non-invasive, and adapted to the current and future technological environment.

### Aim

Since findings from previous research indicate the need for a screening task that assesses multiple language components (Forbes-McKay et al., 2014), we will test digital writing tasks that focus on *motoric* and *cognitive* aspects. To compare the cognitive aspects of text production between the participant groups, the following research questions will be addressed (using the variables listed under each question):

- To what extent do **overall process measures** of cognitively impaired patients differ from those of healthy elderly?

- Time on task.
- Number of characters per minute (fluency).

- To what extent does **pausing behavior in the writing process** of cognitively impaired patients differ from that of healthy elderly?

- Number of pauses per minute and proportion of pause time (to time on task).
- Number of Pause-bursts^1^ (P-bursts) per minute and duration of P-bursts.
- Pause time between words^2^ in general and between words preceding specific word categories.

## Materials and Methods

In a cross-sectional quasi-experiment involving patients with mild cognitive impairment (MCI), mild AD and a healthy control group, we evaluated the main writing process characteristics – as derived from keystroke logging – during the execution of monitored tasks. The study received the ethical approval of the Institutional Review Board ZNA/OCMW (nr. 4157). All participants (and their legal representatives in case of dementia) gave written informed consent for participation in the study.

### Participants

The participants were 30 subjects, distributed over two groups:

(1) A group of 15 patients formally diagnosed with mild cognitive impairment (MCI) due to AD (*n* = 10) and dementia due to AD (*n* = 5), diagnosed according to the NIA-AA criteria. The group consisted of 7 women and 8 men with ages ranging from 62 to 87 years (median age of 74 years).
(2) A group of 15 age- and gender-matched cognitively healthy controls consisting of 7 women and 8 men with ages ranging from 63 to 87 years (median age of 74 years).

A Mann–Whitney *U* test showed no significant difference for age between both groups (*U* = 112.500, *Z* = 0, *p* = 1, *r* = 0). Moreover, all participants underwent a full neuropsychological examination at the time of the study, amongst others consisting of a Mini-Mental State Examination (MMSE; maximum score of 30 with a higher score indicating better cognitive functioning) (Folstein et al., 1975, 1983) and the Geriatric Depression Scale (GDS; maximum score of 30 with a high score indicating significant depressive symptoms) (Sheikh and Yesavage, 1986). The median scores on the MMSE and GDS were, respectively, 29 and 3 for the healthy controls and 26 and 8 for the cognitively impaired patients. The MMSE scores showed, in line with our expectations, a significant difference between both groups (mean ranks of healthy controls and cognitively impaired patients were 19.433 and 11.567, respectively; *U* = 171.500, *Z* = 2.948, *p* = 0.003, *r* = 0.548), as did the GDS scores (mean ranks of healthy controls and cognitively impaired patients were 10.233 and 20.767, respectively; *U* = 33.500, *Z* = −2.776, *p* = 0.006, *r* = −0.534).

### Procedure and Materials

During the experiment, a general questionnaire was completed to gather demographic information and to determine work experience, handedness, computer and typing skills. In addition, the examiner administered the MMSE and the GDS (see section “Participants”) to determine participants’ cognitive status. Finally, participants completed the main tasks consisting of two separate picture description tasks.

**FIGURE 2:**
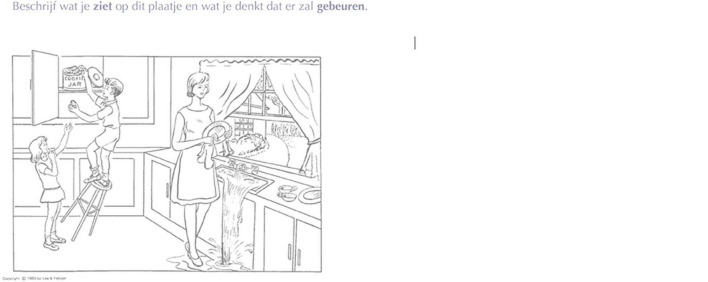
Word template including the Cookie Theft picture.

All participants, when seated at a computer, were instructed to write a short descriptive text for each of two picture description tasks: the Cookie Theft picture from the Boston Diagnostic Aphasia Examination (Goodglass et al., 2001) and the situational drawing from the Dutch version of the Comprehensive Aphasia Test (Swinburn et al., 2005; CAT-NL; Visch-Brink et al., 2014). To avoid order effects, the picture description tasks were counter-balanced. The pictures were included in a MS Word document (see Figure 2). The instruction (translated from Dutch) was as follows: “Describe what you see on this picture and what you think will happen.”

### Data Collection

To observe writing processes on a computer, keystroke logging programs have been developed (Van Waes et al., 2012). These programs collect keyboard and mouse activities during text production. Because they also add a time stamp to each logged event, the resulting log files can be used to reconstruct and describe text production processes. In this study, data were collected with the keystroke logging program Inputlog (Leijten and Van Waes, 2013).

To facilitate the exploration of the cognitive activities during the writing process, the main focus in this study was on pausing behavior. Pauses reflect processes of activating conceptual and linguistic knowledge in order to accomplish specific rhetoric goals (Leijten et al., 2019). Inputlog offers some predefined analyses by default, with the so-called general analysis being the foundation. Apart from some metadata, the general analysis represents the logging process as a fine-grained dataset containing all keystrokes, mouse movements and their respective time stamps in a linear order. Each row in the output represents a keystroke or a mouse action, documented with position and time-based information [key-in and key-out time in milliseconds (ms)] that is used to calculate the pause time between two typing events (e.g., time between two consecutive keystrokes, measured from key-in to key-in). In the context of this study, three other analyses were used: the summary, pause and linguistic analyses (Leijten and Van Waes, 2020). These analyses represent the data of the general analysis on an aggregated level.

As described, the data output from keystroke loggers is mainly based on keystrokes and mouse movements, each captured as isolated events. Moreover, Inputlog aggregates the logged process data from the letter level (keystroke) to the word level as well (Leijten et al., 2015, 2019). Specifically for the linguistic analysis, this allowed us to merge and annotate the word-level output with existing lexica and to analyze these enriched process data with traditional Natural Language Processing (NLP) tools. In this procedure, the logged process data were thus supplemented with different types of linguistic information such as lemmata, part-of-speech tags, syllabification and chunking (see Leijten et al., 2015, 2019, for a more detailed explanation of the linguistic analysis of Inputlog).

#### Variables

##### Overall Process Measures

Time on task and number of characters per minute were calculated for each picture description task. The time on task is the time a participant needs to complete one picture description task. The number of characters per minute are the total number of characters produced (including spaces) divided by time on task in minutes. Here, characters that were typed during the process but that were deleted afterward, are included as well. Data of one participant contained missing values for the overall process measures and was therefore not included in the analyses. Time on task was standardized prior to analysis; number of characters per minute was skewed to the right and therefore normalized by applying a log-transformation.

##### Typing Speed

As typing skills differ between participants, it is necessary to measure the graphomotor latency in fluent text production and isolate these interkey intervals from higher level cognitive pause activities to correctly describe the latter. Hence, a measure based on participants’ interkey latencies, taken from Inputlog’s bigram analysis, was used as a personal indicator of typing speed and added to the analyses (see Table 1). This ensured that the results reflect the cognitive status rather than the motoric typing speed of the participants. The importance of including a graphomotor measure is supported by copy typing studies showing that participants’ interkey intervals non-linearly vary with age, and vary greater in higher age groups (Van Waes et al., 2021).

**TABLE 1:** Overview of mixed-effects models.

Models	Task level
Baseline model	Descriptive or pause variable as dependent variable, typing speed and task as fixed factors, and participant as random factor
Group effect model	Baseline model + group
Part-of-speech effect model	Group effect model + part-of-speech
Interaction effect model	Part-of-speech effect model + group × part-of-speech

##### Pauses and Bursts

Pauses are latencies that last longer than a purely motoric transition between two keys. To account for this, as explained above (see section “Typing Speed”), we added an indicator of typing speed as a personal characteristic to the analyses for each participant. For number of pauses per minute, a technical threshold of 30 ms was also applied in order to filter transition noise of interkey intervals that are created unintentionally (e.g., due to a finger slip when touching a key). One participant’s data contained missing values for number of pauses per minute and proportion of pause time. Number of pauses per minute was right-skewed and therefore normalized; proportion of pause time to time on task was standardized. Pause time between words was normalized as well as standardized.

Finally, a period of active writing between two consecutive pauses (i.e., uninterrupted text production) was considered a P-burst (Chenoweth and Hayes, 2001; Alves and Limpo, 2015; Van Waes and Leijten, 2015). Because the number of P-bursts per minute and duration of P-bursts were chosen to look at fluency rather than low-level cognitive processes, the standard pause threshold of 2,000 ms was applied (Van Waes and Leijten, 2015). Data of one participant contained missing values for number of P-bursts per minute. Prior to analysis, duration of P-bursts was standardized.

### Analysis

First, we examined the effects of group on a number of overall process measures. Second, pause variables were observed at the task level, together with more specific process variables (i.e., pause times between words in general and for word categories) at the word level. Because the data were hierarchical, we used mixed-effects models (Baayen, 2008). For each variable we built models of increasing complexity, with the next model always building on the previous one. Gradually increasing the models’ complexities allowed us to test the impact of each additional factor (see Table 1).

For the overall process measures and the pause variables at the task level only the first two models were built. To test the influence of group on these variables, we first estimated the baseline and the group effect models. The baseline model included task and typing speed as fixed factors and participant as a random factor. In the group effect model, group was added as a fixed effect. To compare the models we used the *likelihood ratio test* and compared the AIC values of the models (Baayen, 2008).

For pause time between words, all four models were built. To test the influences of group and part-of-speech on pause times, we first estimated the baseline, the group effect and the part-of-speech effect models. The baseline model included task and typing speed as fixed factors and participant as a random factor. In the group effect model and the part-of-speech effect model we included group and part-of-speech, respectively. Finally, to test if the influence of group varies between different word categories a group-by-part-of-speech interaction was included in the interaction effect model. Here, we also used the *likelihood ratio test* to compare the AIC values of the models (Baayen, 2008).

Analyses were carried out in R with the packages *lme4*, *car*, and *multcomp* (Hothorn et al., 2008; Bates et al., 2015; Fox and Weisberg, 2019; R Core Team, 2019).

#### Data Reduction

Prior to the analysis of the pause times between words (i.e., variable at the word level, taken from Inputlog’s linguistic analysis), the data set was narrowed down by removing words that included one or more errors (e.g., typing or spelling mistakes). Words revised during the process were also removed, because they made it difficult to estimate how much time should be allocated to the revised word itself as opposed to the one that was being written or planned. Next, cells that contained zero values (e.g., multiple spaces before a word) for between word pauses were removed, and pauses below or above 3.5 times the median absolute deviation (MAD) from the median were considered as outliers (Leys et al., 2013). However, because pause times’ distribution is right-skewed, only outliers above the median were found. Table 2 shows that over 77% of all between word pauses remained after data reduction.

**TABLE 2:** Overview of data reduction for between word pauses.

	Pauses (*n*)	Pauses (%)
Total between word pauses	3,423	100.00%
excl. revisions	3,199	93.46%
excl. errors	2,947	86.09%
excl. zero values	2,929	85.57%
excl. outliers	2,648	77.36%

## Results

We first report on the overall process measures, followed by the pause variables at the task level. Next, we continue with the pause times at the word level. For each dependent variable, model comparisons can be found in Tables 3–6. Tables 7–9 and Supplementary Appendix Table 1 contain the fixed effects estimates for the effects of task, typing speed, group and, if applicable, word categories.

**TABLE 3:** Comparison of the baseline and the group effect models for time on task and number of characters per minute.

	Time on task					Number of characters per minute				
		
	AIC	−2LL	Δ−2LL	Δ df	*p*	AIC	−2LL	Δ−2LL	Δ df	*p*
Baseline model	153.600	143.600				38.246	28.246			
Group effect model	150.010	138.010	5.591	1	0.018	22.531	10.531	17.715	1	<0.001

*AIC, Akaike information criterion; −2LL, deviance; Δ−2LL, chi-square; Δ df, chi-square degrees of freedom; p, p-value.*

**TABLE 4:** Comparison of the baseline and the group effect models for number of pauses per minute and proportion of pause time.

	Number of pauses per minute					Proportion of pause time				
		
	AIC	−2LL	Δ−2LL	Δ df	*p*	AIC	−2LL	Δ−2LL	Δ df	*p*
Baseline model	31.643	21.643				148.200	138.200			
Group effect model	18.474	6.474	15.169	1	<0.001	124.680	112.680	25.520	1	<0.001

*AIC, Akaike information criterion; −2LL, deviance; Δ−2LL, chi-square; Δ df, chi-square degrees of freedom; p, p-value.*

**TABLE 5:** Comparison of the baseline and the group effect models for number of P-bursts per minute and duration of P-bursts.

	Number of P-bursts per minute					Duration of P-bursts				
		
	AIC	−2LL	Δ−2LL	Δ df	*p*	AIC	−2LL	Δ−2LL	Δ df	*p*
Baseline model	206.280	196.280				153.860	143.860			
Group effect model	195.040	183.040	13.238	1	<0.001	129.590	117.590	26.269	1	<0.001

*AIC, Akaike information criterion; −2LL, deviance; Δ−2LL, chi-square; Δ df, chi-square degrees of freedom; p, p-value.*

**TABLE 6:** Comparison of the baseline, the group effect, the part-of-speech effect and the interaction effect models for pause time between words.

	AIC	−2LL	Δ−2LL	Δ df	*p*
Baseline model	5960.200	5950.200			
Group effect model	5956.900	5944.900	5.265	1	0.022
Part-of-speech effect model	5877.200	5851.200	93.755	7	<0.001
Interaction effect model	5885.500	5845.500	5.680	7	0.578

*AIC, Akaike information criterion; −2LL, deviance; Δ−2LL, chi-square; Δ df, chi-square degrees of freedom; p, p-value.*

**TABLE 7:** Estimates of fixed effects for effects of task, typing speed and group on time on task and number of characters per minute.

	Time on task			Number of characters per minute		
		
	Est.	*SE*	*p*	Est.	*SE*	*p*
Intercept*^a^*	–1.122	0.655	0.098	5.522	0.239	< 0.001
Task	0.201	0.156	0.207	–0.061	0.045	0.192
Typing speed	–0.001	0.003	0.815	–0.003	0.001	0.012
Group	0.772	0.328	0.026	–0.566	0.120	< 0.001

*^a^Intercept is Cookie Theft picture, healthy controls.*

**TABLE 8:** Estimates of fixed effects for effects of task, typing speed and group on number of pauses per minute and proportion of pause time.

	Number of pauses per minute			Proportion of pause time		
		
	Est.	*SE*	*p*	Est.	*SE*	*p*
Intercept*^a^*	5.574	0.229	< 0.001	–2.563	0.452	< 0.001
Task	–0.076	0.044	0.098	0.190	0.146	0.202
Typing speed	–0.003	0.001	0.006	0.003	0.002	0.203
Group	–0.490	0.115	< 0.001	1.356	0.225	< 0.001

*^a^Intercept is Cookie Theft picture, healthy controls.*

**TABLE 9:** Estimates of fixed effects for effects of task, typing speed and group on number of P-bursts per minute and duration of P-bursts.

	Number of P-bursts per minute			Duration of P-bursts		
		
	Est.	*SE*	*p*	Est.	*SE*	*p*
Intercept*^a^*	2.291	0.867	0.014	2.422	0.445	< 0.001
Task	0.119	0.260	0.653	–0.028	0.146	0.851
Typing speed	0.001	0.004	0.706	–0.002	0.002	0.336
Group	1.710	0.433	< 0.001	–1.373	0.221	< 0.001

*^a^Intercept is Cookie Theft picture, healthy controls.*

### Overall Process Measures

For time on task, results showed that the group effect model was the best fit when comparing the baseline and the group effect models [*X*^2^ (*Df* = 1) = 5.591, *p* = 0.018; see Table 3]. Hence, there was a statistically significant main effect of group. In Table 7, the coefficient of group shows that patients needed 0.772 standard deviations (which equals 123.836 s) more time to describe the pictures than healthy controls. This means a medium to large effect was found (Sawilowsky, 2009). For the number of characters per minute, the group effect model was the best fit when comparing the baseline and the group effect models [*X*^2^ (*Df* = 1) = 17.715, *p* < 0.001]. This means that also for this variable there was a statistically significant main effect of group. The coefficient of group shows that patients typed 0.566 units (i.e., 108 characters) less per minute (when pure typing speed is already taken into account) than healthy controls.

### Pausing Behavior

For number of pauses per minute, the group effect model was the best fit when comparing the baseline and the group effect models [*X*^2^ (*Df* = 1) = 15.169, *p* < 0.001; see Table 4]. Hence, there was a statistically significant main effect of group. In Table 8, the coefficient of group shows that patients paused 0.490 units (i.e., 102 pauses) less often than healthy controls. For proportion of pause time, results showed that the group effect model was the best fit when comparing the baseline and the group effect models [*X*^2^ (*Df* = 1) = 25.520, *p* < 0.001]. This means that also for this variable there was a statistically significant main effect of group. The coefficient of group shows that pause time in proportion to time on task was 1.356 standard deviations (i.e., 20.589%) higher for patients than for healthy controls, which means a very large to huge effect was found.

For number of P-bursts per minute, the group effect model was the best fit when comparing the baseline and the group effect models [*X*^2^ (*Df* = 1) = 13.238, *p* < 0.001; see Table 5]. Hence, there was a statistically significant main effect of group. In Table 9, the coefficient of group shows that patients’ writing processes contained 1.710 more bursts than healthy controls’. For duration of P-bursts, results showed that the group effect model was the best fit when comparing the baseline and the group effect models [*X*^2^ (*Df* = 1) = 26.269, *p* < 0.001]. This means that also for this variable there was a statistically significant main effect of group. The coefficient of group shows that patients’ P-bursts were 1.373 standard deviations (i.e., 4.926 s) shorter than those of healthy controls, which means a very large to huge effect was found.

#### Pause Time Between Words

**FIGURE 3:**
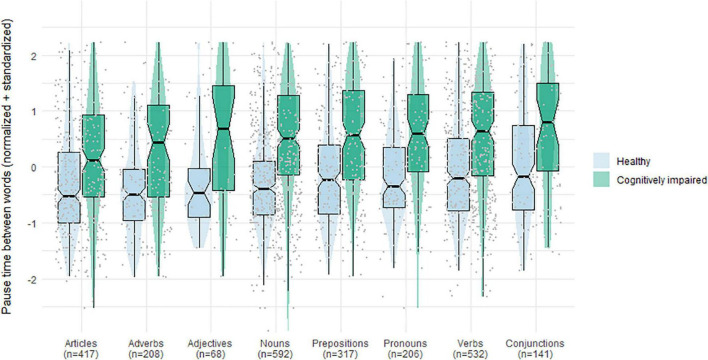
Number of pauses and distribution of pause length per word category for healthy controls and cognitively impaired patients.

Next, we report on pause times between words in general and for specific word categories. Figure 3 shows an overview of the number and distribution of observations per category. With regards to the number of observations, between word pause times of nouns, followed by those of verbs, were the most frequent. Between word pause times of adjectives were the least frequent, followed by conjunctions. In general, observations were less centered around the median for cognitively impaired patients than for healthy elderly. This was especially apparent for pause times preceding adjectives: here observations of healthy elderly were more centered around the median than for any of the other word categories, while the opposite was true for cognitively impaired patients. Moreover, Figure 3 shows that pause times tended to be systematically higher for cognitively impaired patients than for healthy controls.

As mentioned in the section “Analysis,” four models were built by adding an extra factor to the previous model for each new one (see Table 1). We will first compare these models and then discuss the effects based on the best fitting model.

A model comparison comparing the baseline and the group effect models showed a statistically significant main effect of group by pointing to the group effect model as the best fit [*X*^2^ (*Df* = 1) = 5.265, *p* = 0.022; see Table 6]. Moreover, the part-of-speech effect model was a better fit than the group effect model, which pointed to a statistically significant main effect of part-of-speech [*X*^2^ (*Df* = 7) = 93.755, *p* < 0.001]. Finally, no interaction effect between group and part-of-speech was found in a comparison between the part-of-speech effect and the interaction effect models [*X*^2^ (*Df* = 7) = 5.680, *p* = 0.578].

Supplementary Appendix Table 1 contains the fixed effects estimates for the effects of group and part-of-speech in the best fitting model (i.e., the part-of-speech effect model). As for the effect of group, pause times between words increased with 0.496 standard deviations (i.e., 200.683 ms) for cognitively impaired patients compared to healthy controls. As for the effect of part-of-speech, Supplementary Appendix Table 1 only compares the mean of the between word pause of each word category with that of the intercept (i.e., articles). Therefore, a *post hoc* test was run on the effect of part-of-speech to compare all possible word category pairs of means of between word pauses (see Supplementary Appendix Figure 1). This revealed longer pause times between words for prepositions than for articles (*p* = 0.003); for verbs than for articles (*p* < 0.001), adverbs (*p* < 0.001) and nouns (*p* < 0.001); and for conjunctions than for articles (*p* < 0.001), adverbs (*p* < 0.001), adjectives (*p* = 0.006), nouns (*p* < 0.001), pronouns (*p* < 0.001), prepositions (*p* < 0.001) and verbs (*p* = 0.004). The absence of an interaction effect implies that the effects of group and part-of-speech on pause time between words were independent of one another.

**FIGURE 4:**
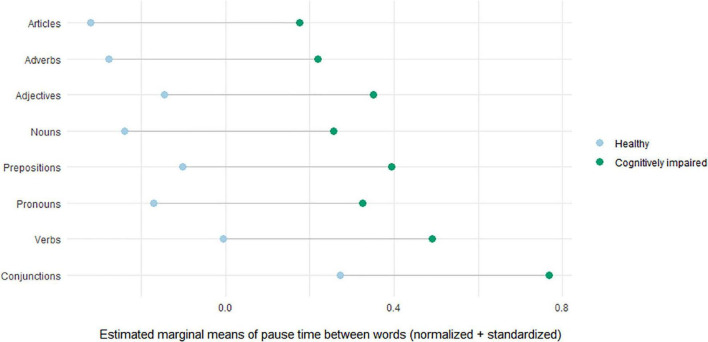
Differences in estimated marginal means of healthy elderly and cognitively impaired patients for pause time between words.

The results are summarized in Figure 4. With regards to group, cognitively impaired patients’ means were consistently higher than those of cognitively healthy elderly. In addition, pause times between words were shortest for articles and longest for conjunctions. Finally, the distance between the means of healthy controls and patients was equal across word categories.

## Discussion

We investigated whether writing process analysis could be a simple and unobtrusive supplementary instrument for identifying (the evolution of) cognitive decline in elderly subjects. Two participant groups were recruited: cognitively healthy elderly and cognitively impaired elderly that belonged to the AD continuum (MCI due to AD and dementia due to AD). Each participant completed a picture description task that consisted of describing two separate pictures of daily situations. The writing process was observed and registered with a keystroke logging tool, Inputlog.

The results of the cross-sectional analyses demonstrate significant effects of cognitive impairment. To start, cognitively impaired patients spend more time on completing the task than healthy controls. Also, patients write less characters per minute. Patients’ typing speed has previously already shown a clear motor slowdown in a comparison with age-matched controls (Van Waes et al., 2017). However, since typing speed was controlled for, this result indicates a decline that is also cognitive in nature.

Results related to pausing behavior show that patients pause less often and that their proportion of pause time to time on task is higher than that of healthy controls. Patients also write in more and shorter bursts (i.e., uninterrupted writing episodes), which indicates that patients have less available working memory capacity to plan and formulate a text chunk (Chenoweth and Hayes, 2003; Hayes and Chenoweth, 2007; Van Waes and Leijten, 2015). In addition, more and shorter bursts reflect a less fluent writing process (Alves and Limpo, 2015). This is supported by the finding that the processes of cognitively impaired elderly are characterized by longer pauses between words, indicating that also these low-level units are cognitively more demanding for them (Leijten et al., 2015).

Because of the importance of linguistic differentiation in studying writing processes (Leijten et al., 2019) and because the production of some word categories is more affected by AD than others (Cappa et al., 1998; Yi et al., 2007) the data were also analyzed with respect to eight word categories. Results show that pauses between words are longer for prepositions than for articles; for verbs than for articles, adverbs and nouns; and for conjunctions than for articles, adverbs, adjectives, nouns, pronouns, prepositions and verbs.

In our view, there are two possible reasons for why pauses between words are longer for verbs than for articles, adverbs and nouns. First, verbs are content words, which makes them semantically more complex than word types that mostly have a grammatical function (here: articles). Second, in comparison with other content words, verbs are also grammatically more complex because in Dutch they need to be conjugated according to tense, person, number, aspect, mood and voice before they can be used in a sentence. These results are also in line with previous studies showing that action naming leads to longer naming latencies than object naming, both in healthy subjects and in AD patients (Szekely et al., 2005; Druks et al., 2006; Mätzig et al., 2009).

The pause differences related to conjunctions can be explained by where this word category is usually positioned in the sentence. Previous research has shown that pauses before lexical units at higher text levels are longer than pauses at lower levels (Medimorec and Risko, 2017). It is therefore likely that pauses between words are longest for conjunctions because they are usually found at the boundary between two clauses or phrases while other word categories are more likely to occur within a clause or a phrase.

Both the differences between word categories and the fact that for each word category a comparable effect is found for both groups, illustrate that linguistic specification of word categories could be an added value in interpreting writing process data. Future research on a larger dataset should confirm this. Moreover, to better determine whether the overall process measures and pause variables can be used to discriminate between the AD continuum group and cognitively healthy elderly, receiver operating characteristic (ROC) curve analyses could be included in a follow-up study (Fawcett, 2006).

### Limitations of the Study

Given the small sample, this study is only exploratory and should be confirmed in a larger cohort. Moreover, this study is performed cross-sectionally at a single moment in time. However, to develop a robust screening tool it should be repeated on several occasions during follow-up to document language change, as described in Forbes-McKay et al. (2013, 2014). We believe that, to date, no longitudinal assessment of the writing process characteristics of AD patients has been carried out to describe the progression of the disease.

Furthermore, there may have been an order effect in the keystroke data because the (neuro)psychological tests (i.e., MMSE and GDS) were administered before the picture description tasks. A study by Vizer et al. (2009) previously showed that keyboard interactions can detect cognitive stress. Hence, if the (neuro)psychological tests induced cognitive stress, it is possible that this was picked up by the typing tasks that followed. However, because we would expect it to affect both groups equally and because this is a cross-sectional study, we believe that it did not influence the results in this paper. For future research, however, it is recommended to start with the typing tasks instead of the (neuro)psychological tests, or to split the experiment into two separate sessions.

### Implications for Further Research

First, although the Cookie Theft picture by Goodglass et al. (2001) has been widely used in previous studies (e.g., Forbes-McKay et al., 2013, 2014), its task validity has, to our knowledge, not been reported. Moreover, the Cookie Theft picture dates back to 1983 and might not be fully adequate anymore in current times. During preliminary analyses, the situational drawing by Visch-Brink et al. (Swinburn et al., 2005; Visch-Brink et al., 2014) proved to have more discriminatory power than the Cookie Theft task (Goodglass et al., 2001). That does not pose a problem for this cross-sectional study because this was accounted for in the mixed-effects models. However, test materials in longitudinal studies should be congeneric and exchangeable (Jöreskog, 1971; Paesen and Leijten, 2019), at least with respect to the most distinguishing variables.

Second, in this paper we only focus on pauses related to eight specific word categories. However, pausing behavior is also dependent on the context in which these word categories occur and on specific features of words or other lexical units. For example, pauses before certain parts-of-speech have shown differences depending on the specific phrases in which they occurred (Leijten et al., 2019). Moreover, both word frequency and length have been shown to influence pause times, with more frequent and shorter words leading to shorter between word pauses before those words (Medimorec and Risko, 2017). Hence, in a follow-up study attention should be given to specific features of lexical units as well as the (linguistic) contexts in which these units occur.

Third, the present study only looks at texts in Dutch. However, specific language properties, such as basic word order [e.g., Subject-Object-Verb in Dutch versus Subject-Verb-Object in English (Bastiaanse and van Zonneveld, 2005)] or aspects of certain word categories [e.g., two definite articles (de/het) in Dutch versus only one in English (the)] may differ between languages. The results in this paper should, therefore, not simply be generalized to other languages and it is recommended that future studies explore the consistency of our findings in languages other than Dutch.

Fourth, the analyses in this paper did not account for changes in the writing processes over the course of the task. However, it could be that participants’ typing and/or pausing behavior changed over time because of factors such as fatigue or stress, due to a learning effect, or because they adapted their strategies to changes in the task situation (Rijlaarsdam and Van den Bergh, 1996; Vizer et al., 2009; Ulinskas et al., 2017; Xu, 2018). To take into account such fluctuations, future studies could treat the writing processes as time series in their analyses (Allen et al., 2016; Pham, 2018).

Fifth, since writing can be considered a combination of graphomotor, sensory and cognitive skills, in a follow-up study we strive to relate writing process data of a motor task (such as a controlled copy task), instead of a measure based on interkey latencies as was done for this study, to a more open writing task. Personal motor characteristics of each participant can then be treated as individual benchmarks for further analysis of the cognitive data. This is important, because interkey intervals differ considerably between participants (Grabowski et al., 2010; Wallot and Grabowski, 2013; Van Waes et al., 2019).

Finally, the process approach presented here should allow online written data to be described and analyzed from a longitudinal perspective. The relative simple applicability also enables writing process researchers to gather a large dataset of normative data from adults of a wide range of ages as to build a normative dataset (Ferguson et al., 2014; Saryazdi et al., 2018; Torrance et al., 2018). Since AD is likely to begin many years before the onset of symptoms (Snowdon et al., 1996; Taler and Phillips, 2008; Blazer and Steffens, 2009; Vermunt et al., 2019), an early, non-intrusive work-up creates added opportunities for screening of the disease and initiation of therapies (Blazer and Steffens, 2009, p. 249).

## Conclusion

This exploratory study indicates that monitoring writing behavior could be a feasible and promising practice in daily clinical practice and could be of added value in the screening of cognitive impairment due to AD. One of the main advantages is that this observation method via keystroke logging is non-intrusive and, because of the possibility of automatization, might also be time-saving and, hence, cost-effective. As the group of computer-literate elderly people grows, writing on a computer will become an even more natural, everyday task, making the technique more broadly deployable and even allowing for remote testing at regular intervals [from participants’ homes; see, for instance, Kaye et al. (2014)]. So, in line with expectations formulated in other research (Snowdon et al., 1996; Taler and Phillips, 2008), writing has a fair potential to be a supplementary tool for observation in the screening and follow-up of AD.

## Data Availability Statement

The data and script used for this study can be found in the following online repository: https://doi.org/10.5281/zenodo.5942516.

## Ethics Statement

This study involving human participants was reviewed and approved by the Institutional Review Board of Hospital Network Antwerp (ZNA). The patients/participants provided their written informed consent to participate in this study.

## Author Contributions

CM: conceptualization, methodology, formal analysis, investigation, data curation, writing – original draft and review and editing, and visualization. ML: conceptualization, methodology, investigation, resources, data curation, writing – original draft and review and editing, supervision, project administration, and funding acquisition. LV: conceptualization, methodology, writing – original draft and review and editing, and supervision. SE: conceptualization, methodology, resources, and writing – review and editing. SD: conceptualization, methodology, formal analysis, writing – review and editing, and supervision. All authors contributed to the article and approved the submitted version.

## Conflict of Interest

The authors declare that the research was conducted in the absence of any commercial or financial relationships that could be construed as a potential conflict of interest.

## Publisher’s Note

All claims expressed in this article are solely those of the authors and do not necessarily represent those of their affiliated organizations, or those of the publisher, the editors and the reviewers. Any product that may be evaluated in this article, or claim that may be made by its manufacturer, is not guaranteed or endorsed by the publisher.

## Footnotes

1 A period of active writing between two consecutive pauses (i.e., uninterrupted text production).

2 A pause time between words consists of the pause between the last character of the previous word and the space, added to the pause between the space and the first character of the next word. Comparable to handwriting, in which a between word pause is considered the time between lifting the pen at the end of a word and putting down the pen to start the next word.
