# Supplementary material for: Cognitive Writing Process Characteristics in Alzheimer’s Disease
Source: Front Psychol. 2022 Jul 11;13:872280. doi: 10.3389/fpsyg.2022.872280 (PMC9311409; doi:10.3389/fpsyg.2022.872280)
Supplement: Supplementary file 1 [file Image_1.pdf]

Supplementary Appendix Figure 1

Differences in estimated marginal means of word categories for pause time between words

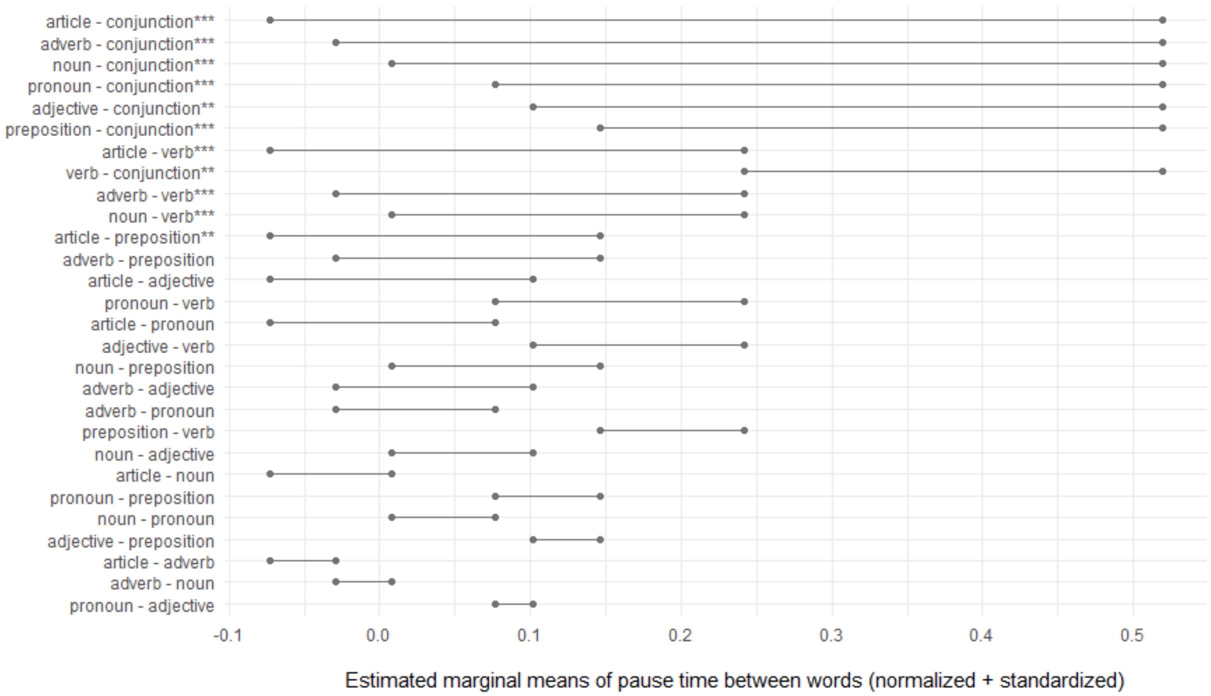

Note. Word category pairs are sorted according to differences in estimated marginal means.

***$p < 0.001$. **$p < 0.01$.
