# Supplementary material for: Cognitive Writing Process Characteristics in Alzheimer’s Disease
Source: Front Psychol. 2022 Jul 11;13:872280. doi: 10.3389/fpsyg.2022.872280 (PMC9311409; doi:10.3389/fpsyg.2022.872280)
Supplement: Supplementary file 2 [file Table_1.pdf]

Supplementary Appendix Table 1

Estimates of fixed effects for effects of task, typing speed, group and word categories on pause time between words

	Est.	*SE*	*p*
Intercept[a]	-1.256	0.344	< .001
Task	0.054	0.032	.087
Typing speed	0.005	0.002	.008
Group	0.496	0.205	.022
Adverb	0.044	0.066	.506
Adjective	0.175	0.102	.085
Noun	0.081	0.049	.102
Preposition	0.219	0.058	< .001
Pronoun	0.149	0.066	.024
Verb	0.315	0.051	< .001
Conjunction	0.593	0.076	< .001

Note. Est. = Estimate; *SE* = Standard Error; *p* = p-value.

[a] Intercept is Cookie Theft picture, healthy controls, articles. Articles were chosen as the intercept because pause times preceding this word category were the shortest.
